# Dosimetric impact of titanium, zirconia, and customized subperiosteal dental implants in head and neck radiotherapy: an *in vitro* study

**DOI:** 10.3389/fonc.2026.1875805

**Published:** 2026-07-22

**Authors:** Kübra Özkaya Toraman, Serap Çatlı Dinç, Can Ilgın, Alp Saruhanoğlu, Özge Kuru Kavak, Levent Demirkol, Gökçe Uçar Alveroğlu, Hatice Bilge Becerir

**Affiliations:** 1 Department of Radiation Oncology, Oncology Institute, Istanbul University, Istanbul, Türkiye; 2 Department of Radiation Oncology, Faculty of Medicine, Gazi University, Ankara, Türkiye; 3 Department of Radiation Oncology, Istanbul Faculty of Medicine, Istanbul University, Istanbul, Istanbul, Türkiye; 4 Department of Oral and Maxillofacial Surgery, Istanbul University Faculty of Dentistry, Istanbul, Türkiye; 5 Department of Medical Physics, Oncology Institute, Istanbul University, Istanbul, Türkiye

**Keywords:** dental implant, dosimetry, head-neck, radiotherapy, subperiosteal implant, titanium, treatment planning system, zirconia

## Abstract

**Background:**

High-density dental implant materials may lead clinically relevant uncertainties in dose calculation and delivery during head and neck radiotherapy (RT). This study aimed to experimentally and computationally evaluate the dosimetric impact of titanium, zirconia, and customized subperiosteal implants.

**Material and methods:**

Three implant types were positioned within artificial bone phantoms (Sawbones^®^) simulating a human mandible. For the subperiosteal group, a patient-specific framework was digitally designed and manufactured. Optically Stimulated Luminescence (OSL) dosimeters were placed at four anatomical locations (distal, buccal, mesial, and lingual) around each implant. Computed tomography (CT) scans (1-mm slice thickness) were obtained, and Intensity-Modulated Radiation Therapy (IMRT) plans were generated using a treatment planning system (TPS) equipped with the AcurosXB (AXB) algorithm with 6-MV photon energy and a target dose of 200 cGy. Statistical analysis was performed using the Wilcoxon Signed-Rank test on dependent groups to compare TPS-calculated and OSL-measured doses.

**Results:**

A significant discrepancy was found between the overall measured and planned doses (p=0.003), with a median dose deviation of -7.8%. Backscatter-induced dose enhancement was systematically detected in the buccal region (up to +1.9%), whereas dose attenuation was predominant in the lingual and mesial regions (up to -14.5%). TPS-calculated maximum point doses revealed substantial hot spots, reaching 228 cGy for zirconia and 217 cGy for subperiosteal implants at the over-the-implant interface. While significant differences were confirmed for the endosseous group (p=0.021), the customized subperiosteal group demonstrated a trend toward a more predictable dosimetric profile (p=0.068) with a lower median deviation (-6.8%) compared to both titanium (-7.8%) and zirconia (-10.5%) endosseous implants.

**Conclusions:**

Dental implant material and design influence dose distribution during RT, with high-density materials such as zirconia associated with greater discrepancies in dose estimation. Customized subperiosteal implants demonstrated a more predictable dosimetric profile in this experimental setting; however, the clinical significance of these findings remains to be established through larger studies incorporating clinical outcome data.

## 1 Introduction

Head and neck cancers represent a global health challenge, with an incidence of approximately 1.1 million new cases annually (1). In the management of these malignancies, head and neck RT continues to play a pivotal role, either as a definitive or adjuvant treatment modality. However, despite its high efficacy in achieving tumor control, RT is associated with significant adverse effects that directly impair patient quality of life. These toxicities are largely influenced by the specific anatomical site of irradiation, making the head and neck region one of the most complex areas to treat due to the requirement for high radiation doses in proximity to critical structures. Consequently, patients often experience debilitating side effects, including xerostomia, radiation-induced caries, and tooth loss.

Currently, dental implants are considered the gold standard for the rehabilitation of partial or complete edentulism. Beyond traditional titanium-based (Z = 22) endosseous implants, zirconia (Z = 40) implants have gained significant clinical popularity, primarily due to their superior aesthetics and favorable biocompatibility at the soft tissue interface (2). Furthermore, in complex cases such as post-oncological treatment defects or severely atrophic jaws where bone volume is insufficient for endosteal placement, customized subperiosteal implants —typically manufactured from medical-grade titanium alloys (Ti-6Al-4V, the same alloy often used for conventional endosseous implants) using additive manufacturing (3D printing) technologies—are gaining renewed interest (3).

The prevalence of dental implants in the general population has risen significantly over the last two decades. Based on large-scale epidemiological data, the prevalence of dental implants is projected to reach as high as 23% by 2026 in certain age groups (4). In particular, head and neck cancer patients often present with extensive tobacco use histories, leading to compromised dental health and a high requirement for dental rehabilitation. Furthermore, for patients undergoing surgery for oral cavity cancers, there is ongoing debate regarding the optimal timing for implant placement: during ablative surgery (primary placement), after surgery but before RT, or in the post-RT period (5). While there is no clear consensus on the ideal treatment sequence, this clinical reality means that a substantial number of patients presenting for radical or adjuvant RT already possess dental implants. Consequently, a precise definition of the radiation interactions associated with various implant materials and types is essential to ensure safe and accurate treatment delivery, minimizing the risk of localized toxicity or target underdosage.

High atomic number and density of dental implants lead to major problems at providing an accurate dose distribution in RT and contouring target volumes and organs at risk (OARs) caused by the artifact in head and neck tumors (6, 7). The extreme Hounsfield Unit (HU) values generated by these materials often exceed the linear calibration limits of standard CT scanners, necessitating extended HU scales for accurate dose calculation. Moreover, these high-density materials interact with the radiation beam, leading to photon attenuation and backscatter (6). Uncontrolled dose enhancements at the implant-tissue interface can clinically trigger severe mucositis and increase the risk of osteoradionecrosis (5, 8, 9).

While the scattering and attenuation patterns of single titanium endosteal implants are relatively well-documented, the impact of zirconia and, more importantly, the emerging technology of customized subperiosteal frameworks remains insufficiently elucidated (10–12). The significantly higher atomic number of zirconia compared to titanium (Z = 22) suggests a more pronounced interaction with the therapeutic beam, potentially leading to more severe dose perturbations at the bone-implant interface. The interconnected nature of subperiosteal frameworks may create a continuous ‘shielding’ or ‘scattering’ effect across a larger anatomical volume compared to discrete endosseous units. Since subperiosteal implants utilize a more extensive, interconnected metal framework compared to isolated endosteal units, their potential for cumulative dose perturbation and artifact generation is theoretically higher, yet clinical data is lacking.

This study aims to evaluate the dosimetric impact of titanium, zirconia, and customized subperiosteal implants by comparing TPS calculations with experimental measurements obtained via OSL dosimetry.

## 2 Materials and methods

### 2.1 Phantom preparation and implant design

Three artificial mandibular models (Sawbones^®^, Vashon, WA, USA), designed to replicate the radiological and physical properties of human cortical bone, were utilized in this study. Firstly, high-resolution CT (Somatom^®^, Siemens, Erlangen, Germany) scans with a thickness of 1mm of the mandibles were obtained, and the data were exported in DICOM format. Then, these DICOM files were converted into STL (Standard Tessellation Language) format, allowing for the customized design of subperiosteal implants for the right posterior region of the mandible. To synchronize the emergence profiles and spatial positioning across all systems, the mandible with the subperiosteal implant was digitally scanned with intraoral scanner (Aoralscan3^®^ Shining 3D, Hangzhou, China) and surgical guides were fabricated (via Exocad^®^ software Darmstadt, Germany) for the other two mandibles (titanium and zirconia). This ensured that all implant systems—subperiosteal, endosseous titanium, and endosseous zirconia—exhibited symmetrical positioning and identical prosthetic emergence profiles.

### 2.2 Implant placement and experimental setup

Implant placement was executed following standard surgical kit protocols using a physiodispenser with controlled settings:

- Group 1: Two titanium-based endosseous implants (Implant Direct^®^, California, USA)
- Group 2: Two zirconia-based endosseous implants (Z-systems^®^, Oensingen, Switzerland).
- Group 3: A custom-made titanium-based subperiosteal implant (CADskills^®^, Ghent, Belgium).

**Figure 1:**
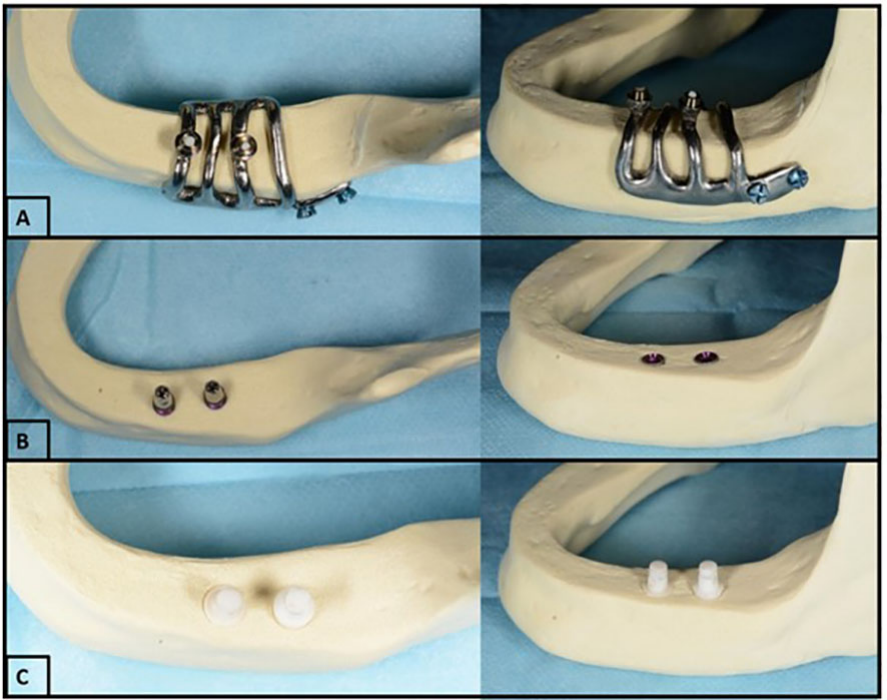
Experimental setups of the different implant types investigated in the study: **(A)** Subperiosteal implant fixed on the mandibular model, **(B)** Titanium endosseous dental implants, and **(C)** Zirconia endosseous dental implants.

To detect dose perturbations around the implants, nanoDot OSL dosimeters (Landauer Inc, Greenwood, IL, USA) were positioned at four critical interfaces: distal, buccal, mesial, and lingual aspects (**Figure 1**).

The prepared mandibles were then placed inside 12.5 cm diameter and 17 cm high plexiglas containers filled with water; these containers served as a soft tissue equivalent material to simulate clinical RT conditions.

### 2.3 Radiological imaging and RT planning

CT imaging was performed using a Philips Big Bore CT scanner (Philips Medical Systems, Highland Heights, OH, USA) with a tube voltage of 120 kVp and 1-mm slice thickness. The CT datasets were then imported into the Eclipse TPS (v15.6, Varian Medical Systems, USA). The HU values for all three materials were measured and recorded. The CT datasets were then imported into the Eclipse TPS (v15.6, Varian Medical Systems, USA). IMRT plans were generated using 6 MV photon beams to deliver a prescribed dose of 200 cGy to target volumes representing tumor tissue. Dose calculations were performed using the Acuros XB (AXB) algorithm. Dose calculations were performed using a grid size of 2 mm with heterogeneity correction enabled. Extended CT-to-electron density conversion table available in the TPS was applied. Acuros XB (AXB) is a deterministic dose calculation algorithm based on the numerical solution of the linear Boltzmann transport equation, providing accurate dose estimation in heterogeneous tissues with variable electron density.

**Figure 2:**
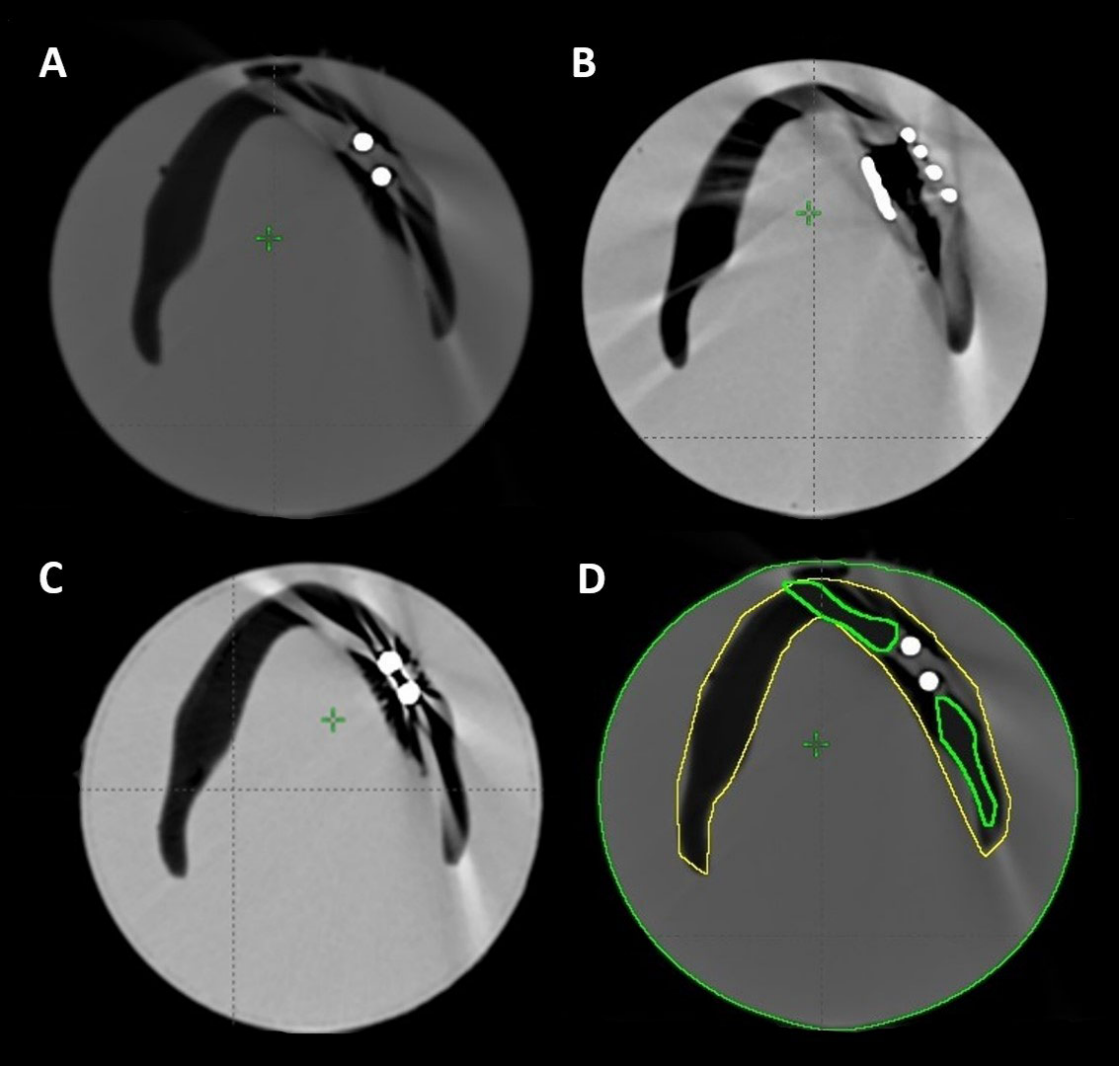
Representative axial CT slices demonstrating relative artifact severity and density management across different implant configurations: **(A)** Titanium endosseous implant, **(B)** Customized subperiosteal implant framework, **(C)** Zirconia implant exhibiting the most severe streak artifacts with a peak value of 38,059 HU, and **(D)** Visual demonstration of the artifact management protocol, where artifact-induced artificial air cavities in the adjacent tissue were manually contoured and assigned normal tissue HU values to ensure dosimetric accuracy.

Implant structures were manually contoured on the CT images based on their visible boundaries. Metal-induced artifacts, particularly around zirconia implants, were visually assessed during contouring. No dedicated metal artifact reduction (MAR) technique was applied, allowing evaluation of the dosimetric impact under standard clinical planning conditions. The artificial air regions within the adjacent soft tissues caused by the artifacts were manually contoured, and HU values corresponding to normal tissue were assigned to prevent artificial underdosages. Furthermore, since these high-density dental materials cannot be explicitly selected as pre-defined configurations within the TPS, no manual HU overrides were assigned to the implant cores themselves. To maintain consistency among groups, contouring was performed using identical window-level settings and reviewed by an experienced radiation oncologist and medical physicist. All dose calculations were performed on the original CT datasets using the extended CT-to-electron density conversion table available in Eclipse TPS to accurately reflect routine clinical treatment planning conditions (**Figure 2**).

### 2.4 Irradiation and dosimetric measurement

The phantom setup was irradiated using a Varian TrueBeam linear accelerator with 6 MV photon energy (Varian Medical Systems, USA). No manual density override was performed for implant materials. Linear accelerator dose calibration: The output dose calibration of the linear accelerator was performed using the IAEA TRS 398 protocol, with a maximum dose depth of 10x10 cm² and an SSD of 100 cm² at 1 cGy/1 MU (13). The dose rate was 300 cGy/min.

OSL dose calibration: 18 dosimeters with the same sensitivity were selected for calibration. Each nanodot was placed at the center of a 10x10 cm² area on a 5 cm thick solid phantom. The calibration setup was created by placing a 1 cm bolus and a 0.6 cm solid phantom (dmax=1.6 cm) on top of the placed dosimeter. For calibration, the dosimeters were irradiated with low doses of 0 cGy, 1 cGy, and 5 cGy, and high doses of 50 cGy, 100 cGy, 150 cGy, 300 cGy, 500 cGy, and 800 cGy using 6 MV high-energy photons. The dosimeters were read with a MicroStar reader (Landauer Inc, Greenwood, IL) three times at least 10 minutes after irradiation, and the average of the doses was taken to create a calibration curve. The overall uncertainty of OSL dosimetry, including calibration, energy dependence, and positioning, was estimated to be within ±5%.

The phantoms were irradiated with 6 MV photons at a dose of 2 Gy using the IMRT technique, according to the prepared plans. Following irradiation, dose values ​​were read from the OSL dosimeters using a MicroStar reader. To ensure dosimetric reliability and reproducibility, each measurement point was subjected to three repeated irradiations.

### 2.5 Statistical analysis

Differences between the theoretical doses calculated by the TPS and the experimental measurements obtained via OSL dosimeter were analyzed using Stata version 15.1 (StataCorp, 4905 Lakeway Drive, College Station, TX 77845, US)). After testing the normality assumption, the non-parametric Wilcoxon Signed-Rank test was applied to evaluate the significance of differences between the TPS-calculated and OSL-measured doses. For all analyses, a p-value of < 0.05 was considered statistically significant.

Due to the complexity of phantom preparation and repeated irradiation measurements, the sample size was limited. The study was designed as a pilot study.

## 3 Results

In this study, the dosimetric effects of three distinct dental implant materials (Titanium, Zirconia, and Subperiosteal) were analyzed by comparing TPS-calculated doses with those obtained experimentally by using OSL dosimetry (**Table 3**).

### 3.1 Radiological findings and HU values

HU analysis performed on CT images revealed significant differences between the materials.

Due to these metal implants create artifacts on the adjacent soft tissue, they may not show the relative electron density. Severe metal artifacts were observed, particularly in the zirconia group, which introduced challenges in contouring the adjacent soft tissues.

### 3.2 Comparative dosimetric analysis

OSL measurements and TPS-calculated doses recorded at four critical interfaces (Distal, Buccal, Mesial, and Lingual) are comparatively presented in **Table 1**. The overall analysis of 12 measurement points revealed a significant discrepancy between the measured and planned doses (p=0.003), with a median dose deviation of -7.8% (range: -14.5% to +1.9%). The median measured dose across all groups was 184.5 cGy, compared to the TPS median of 200 cGy.

**Table 1:** Comparison of TPS-calculated and OSL-measured dose values among different implant materials.

Material	Dosimetric measurement locations	OSL-measured dose* (cGy)	TPS-calculated dose (cGy)
Subperiosteal implant	Distal	193 ± 2	200
	Buccal	188 ± 2	208
	Mesial	188 ± 1	200
	Lingual	185 ± 3	200
Titanium dental implant	Distal	183 ± 9	198
	Buccal	205 ± 5	209
	Mesial	182 ± 6	199
	Lingual	184 ± 9	200
Zirconia dental implant	Distal	177 ± 1	200
	Buccal	213 ± 2	209
	Mesial	171 ± 8	200
	Lingual	181 ± 3	200

*Data are presented as mean ± standard deviation (SD) of n=3 independent measurements per point.

When stratified by implant type, distinct dosimetric patterns emerged. The endosseous group (titanium and zirconia) showed a statistically significant discrepancy between OSL measurements (median: 182.5 cGy) and TPS calculations (median: 199.5 cGy) (p=0.021), resulting in a median deviation of -8.3%. In contrast, the customized subperiosteal group demonstrated descriptive trend toward a more predictable dosimetric profile, with a non-significant difference between OSL-measured (median: 188 cGy) and TPS-calculated (median: 200 cGy) doses (p=0.068) and a lower median deviation of -6.8%.

### 3.3 Material-specific and anatomical variations

Material-specific descriptive analysis showed that zirconia exhibited the most substantial dose underestimation by the TPS, with a median percentage difference of -10.5% (p=0.144). For titanium configurations, both endosseous and subperiosteal designs showed median deviations of -7.8% and -6.8% respectively (p=0.068).

Regarding anatomical locations, dose attenuation (shadow effect) was most prominent in the mesial (median difference: -8.5%) and lingual (median difference: -8.0%) regions. Conversely, the buccal interface showed the least deviation (median: -1.9%) and was the only region where positive dose enhancement (backscatter) was recorded, specifically reaching +1.9% in the zirconia group.

### 3.4 Impact of distance and positioning (Ta and Tb points)

**Figure 3:**
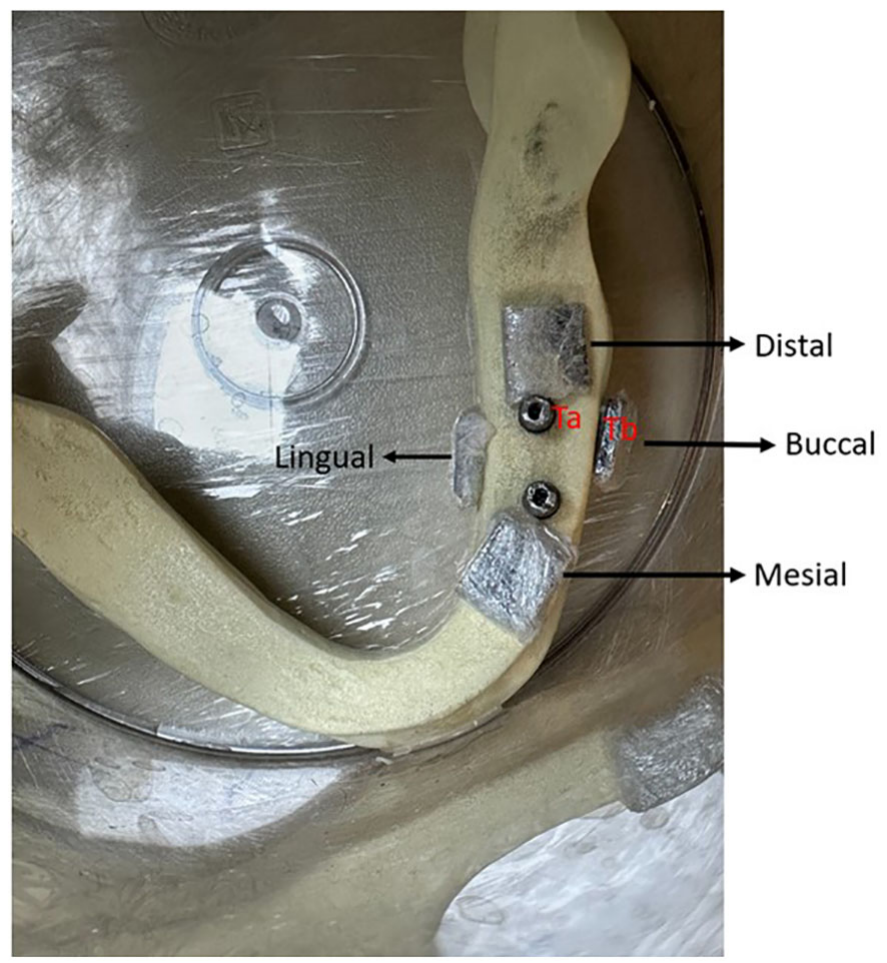
Anatomical placement points of nanoDot OSL dosimeters. Measurements were recorded at four specific interfaces for each implant group: mesial, distal, buccal, and lingual.

As shown in **Table 2**, the TPS-calculated maximum point doses identified substantial “hot spots” at the over-the-implant interface (Ta point) (**Figure 3**). Zirconia reached the highest peak at 228 cGy, followed by titanium (220 cGy) and subperiosteal implants (217 cGy). However, these peak doses were not fully captured during experimental measurements due to the physical dimensions of the dosimeters (nanoDot) and their placement on the cortical bone (**Table 4**). Consistent with the literature, the backscatter effect is known to be limited to a 2-mm range; this distance-dependent decay is the primary reason for the discrepancy between the theoretical peak calculations and experimental measurements.

**Table 2:** Maximum point doses calculated by TPS.

Material	Over-the-implant (cGy)	Buccal Ta* (cGy)	Buccal Tb* (cGy)
Subperiosteal	217	208	208
Titanium	220	210	209
Zirconia	228	214	209

*Ta, Point A at the buccal interface; Tb, Point B at the buccal interface.

**Table 3:** Descriptive statistics.

Category	Parameter	Group	n	Median	IQR	Min	Max
Overall		Measured (cGy)	12	184.5	9	171	213
		TPS (cGy)	12	200	4	198	209
		Difference (cGy)	12	–15.5	10	–29	4
		% Difference	12	–7.8	4.8	–14.5	1.9
By implant type	Endosseous	Measured (cGy)	8	182.5	15.5	171	213
		TPS (cGy)	8	200	5	198	209
		Difference (cGy)	8	–16.5	11.5	–29	4
		% Difference	8	–8.3	5.8	–14.5	1.9
	Subperiosteal	Measured (cGy)	4	188	4	185	193
		TPS (cGy)	4	200	4	200	208
		Difference (cGy)	4	–13.5	8	–20	–7
		% Difference	4	–6.8	3.8	–9.6	–3.5
By material	Titanium (endosseous)	Measured (cGy)	4	183.5	12	182	205
		TPS (cGy)	4	199.5	6	198	209
		Difference (cGy)	4	–15.5	7	–17	–4
		% Difference	4	–7.8	3.5	–8.5	–1.9
	Titanium (subperiosteal)	Measured (cGy)	4	188	4	185	193
		TPS (cGy)	4	200	4	200	208
		Difference (cGy)	4	–13.5	8	–20	–7
		% Difference	4	–6.8	3.8	–9.6	–3.5
	Zirconia (endosseous)	Measured (cGy)	4	179	23	171	213
		TPS (cGy)	4	200	4.5	200	209
		Difference (cGy)	4	–21	18.5	–29	4
		% Difference	4	–10.5	9.2	–14.5	1.9
By anatomical location	Buccal	Measured (cGy)	3	205	25	188	213
		TPS (cGy)	3	209	1	208	209
		Difference (cGy)	3	–4	24	–20	4
		% Difference	3	–1.9	11.5	–9.6	1.9
	Distal	Measured (cGy)	3	183	16	177	193
		TPS (cGy)	3	200	2	198	200
		Difference (cGy)	3	–15	16	–23	–7
		% Difference	3	–7.6	8	–11.5	–3.5
	Lingual	Measured (cGy)	3	184	4	181	185
		TPS (cGy)	3	200	0	200	200
		Difference (cGy)	3	–16	4	–19	–15
		% Difference	3	–8.0	2	–9.5	–7.5
	Mesial	Measured (cGy)	3	182	17	171	188
		TPS (cGy)	3	200	1	199	200
		Difference (cGy)	3	–17	17	–29	–12
		% Difference	3	–8.5	8.5	–14.5	–6.0

**Table 4:** Wilcoxon signed-rank test: measured dose vs. TPS dose.

Stratum	Subgroup	n	z	p
Overall		12	–2.944	**0.003**
By implant type	Endosseous	8	–2.313	**0.021**
	Subperiosteal	4	–1.826	0.068
By material	Titanium (endosseous)	4	–1.826	0.068
	Titanium (subperiosteal)	4	–1.826	0.068
	Zirconia	4	–1.461	0.144
By anatomical location	Buccal	3	–0.816	0.414
	Distal	3	–1.604	0.109
	Lingual	3	–1.604	0.109
	Mesial	3	–1.604	0.109

Bold values indicate statistically significant differences (p < 0.05).

## 4 Discussion

This study investigated the dosimetric behavior of titanium, zirconia, and customized subperiosteal implants under 6 MV photon irradiation using both AXB -based TPS calculations and experimental OSL dosimetry. The results demonstrate that while all high-density materials influence dose distribution, the endosseous group—driven largely by the high atomic number of zirconia—showed a statistically significant discrepancy between planned and measured doses (p=0.021). Although zirconia implants demonstrated the largest median dose deviation in the present dataset (-10.5%), this observation should be interpreted as a descriptive trend rather than a statistically confirmed material-specific effect, given the limited sample size and the absence of statistical significance (p=0.144).

The extremely high peak density measured for zirconia implants (37,744 HU) resulted in severe metal artifacts on CT imaging, significantly exceeding the values observed for standard titanium (11,263 HU) and subperiosteal (9,351 HU) materials. In head and neck RT, accurate target delineation is critically dependent on reliable CT-based electron density mapping. Metallic artifacts are known to compromise contouring accuracy and may introduce uncertainties in heterogeneity correction within TPS algorithms (14, 15). Our findings confirm that zirconia, due to its higher effective atomic number, generates more substantial beam hardening and streak artifacts compared to titanium-based alloys. Clinically, this may lead to contouring inaccuracies around mucosal surfaces and cortical bone adjacent to the implant, potentially affecting both tumor coverage and OAR sparing.

The severe streak artifacts generated by zirconia not only mask the true anatomical boundaries but also systematically distort the automated electron density assignments within the TPS by creating artificial ‘dark’ and ‘bright’ bands. This physical distortion frequently requires the radiation oncologist and medical physicist to utilize manual contouring adjustments, as the calculation algorithm cannot accurately compute dose profiles through clinically compromised HU maps. Therefore, when zirconia is present, the use of advanced artifact reduction algorithms or manual density overrides is strongly recommended to ensure dosimetric integrity.

The attenuation observed distal to the implant structures reflects increased photon absorption and scattering within these high-density materials. Previous *in vitro* investigations have reported dose reductions behind metallic restorations ranging from 5% to 15%, depending on beam energy and geometry (14). In the present study, despite a prescribed dose of 200 cGy, experimental OSL measurements in the distal, mesial, and lingual regions showed marked reductions, particularly in the zirconia group (ranging from 171 to 181 cGy). Statistical analysis via the Wilcoxon Signed-Rank test confirmed that these discrepancies were significant for the endosseous group (p=0.021). This indicates a systematic overestimation of the dose by the AXB-based TPS when calculating behind high-Z materials. From a clinical perspective, this attenuation becomes critical when the target volume or high-risk areas are located posterior to the implant site. Even a moderate underdosage in regions potentially harboring microscopic disease may theoretically compromise local control, especially in definitive head and neck RT where precision is paramount.

More clinically concerning is the interface dose enhancement caused by backscatter radiation. In our study, the buccal measurements demonstrated a localized increase in surface dose, particularly in the zirconia group, where OSL readings at the Tb point showed a median increase of approximately 4% above the TPS-predicted values. This escalation is consistent with the physical properties of backscatter radiation from high-Z materials, which is typically confined to a short range of approximately 1–2 mm (14) Consequently, the actual magnitude of dose enhancement at the immediate mucosa–implant interface may be greater than experimentally detected. However, this discrepancy between TPS-calculated interface doses and OSL measurements requires careful interpretation. The TPS-derived values at the Ta point represent localized peak doses occurring immediately adjacent to the implant surface and therefore reflect theoretical maximum dose perturbations. In contrast, nanoDot OSL dosimeters measure the absorbed dose averaged over their finite sensitive volume. Consequently, the experimentally recorded doses are not expected to reproduce highly localized dose peaks with submillimeter spatial extent. Because backscatter-induced dose enhancement is generally confined to the first 1–2 mm from the implant interface, spatial averaging within the OSL detector may partially smooth these gradients, resulting in an underestimation of the true microscale peak dose. Therefore, TPS-predicted hot spots and OSL-measured doses should be regarded as complementary rather than directly equivalent representations of the interface dose distribution. Although the observed dose enhancement was spatially confined to the implant–tissue interface, such localized dose perturbations may represent a potential contributing factor to radiation-induced tissue reactions. However, the present *in vitro* study was not designed to evaluate clinical toxicity, and therefore no direct association with mucositis, osteoradionecrosis, or implant failure can be established.

The discrepancy between AXB-calculated build-up doses (up to 228 cGy at the Ta point) and experimentally measured values deserves careful consideration. Comparative studies have demonstrated that Monte Carlo simulations provide more accurate representation of dose perturbations in the presence of metallic heterogeneities (15–17). In our results, while the TPS predicted ‘hot spots’ up to 228 cGy at the immediate implant interface (Ta point), the OSL dosimeters—placed slightly further on the bone surface (Tb point)—captured a more attenuated dose increase. Furthermore, the finite dimensions of the nanoDot OSL dosimeters likely resulted in spatial averaging, which may have further masked the true peak of the interface dose. Whereas the increase in dose could be calculated using the TPS, it could not be observed in the experimental results. Because the volume of the nanoDot OSL dosimeters was not sufficient for measuring scatter and backscatter, the scatter and backscatter radiation measured with nanoDot OSL dosimeters also could not be observed. Consequently, the actual magnitude of dose enhancement at the immediate mucosa–implant interface may be even greater than experimentally detected.

When comparing implant types, zirconia consistently demonstrated the greatest perturbation, followed by traditional titanium and subperiosteal implants. Notably, despite its more extensive and interconnected framework, the customized subperiosteal implant exhibited lower HU values and comparatively reduced dose alterations—a finding that did not reach statistical significance (p=0.068), reflecting a clinical trend. This suggests a potentially more predictable dosimetric profile that warrants cautious interpretation due to the small sample size. This is particularly relevant in oncologic rehabilitation planning. In patients where adjuvant RT is anticipated, the choice of implant material should incorporate radiophysical considerations alongside mechanical stability and esthetics. The growing popularity of zirconia for esthetic reasons must therefore be carefully balanced against its higher radiodensity and the potential for significant dosimetric consequences in the irradiated patient.

The clinical implications of these findings underscore the necessity of multidisciplinary coordination in head and neck cancer management. While pre-RT dental assessment typically focuses on extraction decisions, our results suggest that the architectural design and material composition of existing implants—particularly high-density zirconia—significantly influence microscale dose distribution. The finding that subperiosteal frameworks demonstrated a trend toward a more predictable dosimetric profile (p=0.068) compared to the significant discrepancies observed in endosseous implants (p=0.021) provides a new perspective on material-specific risks. Specifically, the substantial dose underestimation by the TPS in zirconia and titanium groups could lead to unintended ‘cold spots’ in target volumes or ‘hot spots’ at the buccal interface due to backscatter. Therefore, optimization strategies such as beam angle modification, artifact reduction algorithms, or density override adjustments within the TPS may help mitigate unintended dose heterogeneities.

Several limitations of this study should be acknowledged. Crucially, as a pilot *in vitro* investigation, this study utilized a relatively small number of measurement points (n=12 overall, with n=4 per specific implant configuration). Due to the complexity of phantom preparation and repeated irradiation measurements, the number of samples was restricted, which inherently restricts the statistical power to detect subtle differences. Additionally, while the artificial bone phantoms used (Sawbones^®^) replicate the physical properties of cortical bone, this *in vitro* model does not fully capture the complex anatomical heterogeneity of living tissues, such as varying mucosal thickness and vascularization, which are critical in the development of osteoradionecrosis. An additional limitation of this study is that no dedicated MAR technique was used during CT acquisition or image reconstruction. Consequently, metal-induced artifacts, particularly around zirconia implants, may have affected contouring accuracy and electron density assignment within the TPS. Future studies should investigate whether advanced MAR algorithms can improve dose calculation accuracy and reduce uncertainty in the presence of high-density dental materials. Furthermore, dosimetric evaluations were limited to a 6-MV photon energy and a single IMRT technique. In clinical head and neck RT, 6-MV photon energy is widely preferred as the standard beam energy because it provides adequate depth-dose characteristics for tumor coverage while avoiding the risk of neutron contamination associated with higher photon energies. Regarding the planning modality, IMRT was specifically selected over Volumetric Modulated Arc Therapy (VMAT) to allow for stricter manual optimization of beam angles, effectively minimizing direct irradiation through high-density implant cores. However, alternative modalities like VMAT or alternative energies may demonstrate different interaction profiles, such as altered forward and backward scattering ratios. Future investigations integrating advanced dose calculation algorithms and direct clinical toxicity correlations would strengthen the biological interpretation of the observed interface dose perturbations. In line with this, an interesting future direction would be to investigate whether the application of customized intraoral protective devices, radiation splints, or tissue-equivalent spacers could reduce the contribution of secondary electrons and backscatter radiation to adjacent mucosal tissues around high-density dental implants. Such protective interfaces could potentially mitigate localized dose enhancement at the implant–mucosa boundary, particularly for zirconia implants where the strongest backscatter effects were observed.

Although the observed dose perturbations were spatially confined, their localization at the implant–mucosa and implant–bone interface suggests potential relevance for radiation-induced toxicity patterns in head and neck patients. Even limited interface dose escalation may contribute to mucosal inflammation, impaired healing, or increased susceptibility to osteoradionecrosis in high-dose regions, especially given the significant discrepancies found in endosseous implants. Conversely, the more predictable dosimetric profile of customized subperiosteal frameworks suggests that architectural design could be a mitigating factor in treatment planning. These findings highlight the importance of incorporating specific implant material characteristics and design considerations into pre-RT dental assessments and multidisciplinary treatment planning to ensure both oncological safety and peri-implant tissue health in head and neck carcinoma patients.

## Conclusion

This study demonstrates that dental implant material and structural design influence dose distribution during head and neck RT. Among the investigated materials, zirconia implants showed the largest descriptive discrepancy between calculated and measured doses. However, due to the limited sample size and lack of statistical significance, these findings should be considered preliminary and hypothesis-generating rather than conclusive evidence of material-specific dosimetric differences. While customized subperiosteal implants showed a trend toward a more predictable dosimetric profile, these findings require confirmation in larger studies. Incorporating material-specific considerations into RT planning may improve dose accuracy and reduce uncertainty in patients with dental implants.

## Data Availability

The raw data supporting the conclusions of this article will be made available by the authors, without undue reservation.
